# Safety of fasting in diabetic and non-diabetic patients with stable chronic kidney disease during Ramadan

**DOI:** 10.12669/pjms.40.4.7989

**Published:** 2024

**Authors:** Anita Haroon, Musarrat Riaz, Bushra Rabbani, Ammarah Saeed

**Affiliations:** 1 Dr. Anita Haroon, MRCP , FCPS (Nephrology), FRCP Assistant Professor, Department of Nephrology, Baqai Medical University, Karachi, Pakistan; 2 Dr. Musarrat Riaz, FCPS (Med), FCPS (Endocrinology). Associate Professor, Department of Medicine, Baqai Medical University, Karachi, Pakistan; 3 Dr. Bushra Rabbani, FCPS. Assistant Professor, Department of Medicine, Baqai Medical University, Karachi, Pakistan; 4 Dr. Ammarah Saeed, FCPS. Assistant Professor, Department of Medicine, Fazaia Medical College, Karachi, Pakistan

**Keywords:** Ramadan, Fasting, Stable CKD, eGFR

## Abstract

**Objective:**

To assess the safety of fasting in diabetic and non-diabetic patients with stable CKD during Ramadan.

**Methods:**

This prospective observational study was conducted at three secondary and tertiary care hospitals of Karachi during the month of Ramadan from 25^th^ March to 7^th^ May 2022. Patients who met the eligibility criteria were assessed pre-Ramadan, and their baseline blood pressure, serum urea, creatinine, electrolytes, uric acid, and estimated glomerular filtration rate (eGFR) were documented and tracked over the course of Ramadan and up to one week post-Ramadan. Deterioration in renal functions (defined as a rise of ≥30% in serum creatinine from the baseline or the decline of ≥25% in eGFR from the baseline) was observed during the month of Ramadan.

**Results:**

A total of 68 patients (34 males and 32 females) with the mean age 58.7±12.16 years were included in the study. Out of 66 patients, 38 were diabetic and 28 were non diabetic. Majority 23(34.8%) were stage-3a and 23(34.8%) were stage-4 followed by stage-3b in 14(21.2%), stage-2 in 3(4.5%) and stage-5 in 3(4.5%) patients respectively. Statistically significant improvement in pre and post Ramadan values of systolic and diastolic blood pressure, serum creatinine and uric acid levels were found in both diabetic and non-diabetic group (P value <0.0001).

**Conclusion:**

Our study shows no worsening of renal functions in both diabetic and non-diabetic patients with stable CKD who intended to fast.

## INTRODUCTION

Ramadan is the 9^th^ month of the Islamic calendar and Muslims practice obligatory fasting during this month, which may range from 12 to 16 hours and sometimes more in some countries depending upon their geographical location and season.^1^ However, those who are sick, old or suffering from chronic diseases in whom there is a chance of aggravation of the preexisting illness due to long hours of fasting, are some situations in which Islam offers relaxation.^2^

As per Global Kidney Health Atlas Survey 2017(GKHA) around 10% of the people are suffering from chronic kidney disease (CKD) worldwide. Middle and low income countries (M&LIC) are mostly affected with the huge burden of CKD having meagre resources to deal with the disease complications.^3^ People with CKD are at increased risk of developing complications during fasting such as low blood pressure, electrolytes imbalance, hypoglycemia, hyperuricemia and dehydration. Similarly chances of urinary tract infections and nephrolithiasis increases if good hydration is not maintained after breaking the fast.^4^ These patients are categorized as having very high risk of complications if they fast. Therefore, it can be challenging for clinicians to manage chronic illnesses like CKD with long hours of fasting and safety concern arise when patients insist on fasting against medical advice.^5^

A few studies have been done in many countries to evaluate the safety of fasting in people with CKD with conflicting results. The disparity in results may be because most of the studies were under powered with small number of patients and were done in those parts of the world who have differences in their climatic conditions.^6^ Healthcare professionals are often asked by their CKD patients who intend to fast about the safety of fasting however there are no clear cut answers to their queries. Still physicians used to give advices to their patients mostly on individual basis and on their experiences due to insufficient large cohorts and diversity of results on this topic. No defined protocols are available to address this issue.

In recent IDF-DAR guidelines, individuals with CKD from stage three to five are considered high risk and discouraged from fasting however those who still prefer to fast needs careful monitoring on weekly basis.^7^ As data about safety of fasting in people with CKD is scarce specially from our part of the world, hence we planned this study to assess the safety of fasting in stable CKD patients both diabetic and non-diabetic.

## METHODS

This prospective observational study was conducted at the outpatient departments of three hospitals namely OMI Hospital, Imam Clinic and Fatima Hospital, Baqai Medical University, Karachi from 3^rd^ April to 2^nd^ May 2022 (with total 14 hours of fast per day).

### Ethical Approval

Data collection was done after the approval of ethical review committee from Baqai Medical University with reference number of BMU-EC/05-2021. Permission was also obtained from other institutions from where data was collected.

All the patients ≥18 years of age and with stable CKD (i.e. no or <10% fluctuations in serum creatinine in last three months) from stage one to five who intended to fast during the month of Ramadan were included in the study. Patients who intended to fast were counselled in detail about the potential risk of fasting on one to one basis. Patients were counselled for proper intake of liquids in non-fasting hours to maintain their hydration status, detailed dietary counselling were also done in order to avoid electrolytes imbalances. Patients were called for assessment and follow up thrice in a month. Diabetic patients were also counselled by diabetic educators for proper sugar monitoring and diet in order to prevent hyper and hypoglycemic events or if at any point they had to break their fast. Patients were also inquiring for urinary tract infections during the whole period and were asked to contact respective health care provider immediately in case of any adverse event. Patients with a history of recent cerebrovascular disease, malignant hypertension, kidney transplant recipients, having acute infection as well as those who were on dialysis were excluded.

Basic demographic details and medical history were recorded in the pre-defined questionnaire. All the study participants were seen thrice in OPD during the study period, Before Ramadan, two weeks after commencing the fast and one week after the end of the month of Ramadan. Blood pressure (BP) was recorded at every visit. Laboratory investigation including urea, serum electrolytes serum creatinine (S.Cr.), eGFR and uric acid (UA) were done at each visit. Patients were also asked if they had to break their fast due to any problem during the whole study period. Appropriate changes in the treatment of diabetic patients were done as per international recommendations of IDF - DAR guidelines.

Deterioration in renal functions (defined as a rise of ≥30% in serum creatinine from the baseline or the decline of ≥25% in eGFR from the baseline) was observed during the month of Ramadan. CKD EPI equation 2021 was used to calculate the eGFR (ml/minute).

### Statistical Analysis

Data was analyzed by using SPSS version 20. Continuous variables were reported as mean ± standard deviation while categorical variables were reported as frequency (percentage). Paired T-test, Student’s T-test and Chi squared test were applied for comparison. P-value <0.05 was set as statistical significance.

## RESULTS

A total of 68 patients were included in the study however, two patients developed UTI during the study and were asked not to fast hence excluded from the study. Mean age of the participants was 58.7±12.16 years, among them 34(51.5%) were males and 32(48.5%) were females.

Study participants were divided into different stages of CKD out of which majority 23(34.8%) were stage-3a and 23(34.8%) were stage-4 followed by stage-3b in 14(21.2%), stage-2 in 3(4.5%) and stage-5 in 3(4.5%) patients respectively (Table-I). Mean Pre Ramadan baseline values of Systolic and Diastolic BP was 131.36±12.14 and 76.21±6.51 respectively. Pre Ramadan mean serum creatinine was 2.22±0.84, e GFR was 38.05±14.92 and Uric Acid was 6.38±1.54 (Table-II). However, in post Ramadan assessment statistically significant improvement was found in mean systolic and diastolic blood pressure i.e. 125.09±7.23 and 72.42±4.5 respectively. Also, improvement in post Ramadan values of serum creatinine and uric acid levels were also noticed (P value <0.0001) as mentioned in (Table-II).

**Table-I:** Characteristics of studied participants.

Parameters	n(%)
n	66
Male	34(51.5%)
Female	32(48.5%)
Age (years)	58.7±12.16
***CKD stage of baseline***	
2	3(4.5%)
3a	23(34.8%)
3b	14(21.2%)
4	23(34.8%)
5	3(4.5%)
***Comorbids***	
DM	38(57.6%)
HTN	47(71.2%)
IHD	3(4.5%)
CVA	0(0%)
Others	1(1.5%)
Duration of CKD	4.81±2.51
***Greater than or equal to 30% rise in serum creatinine from baseline***	
No	66(100%)
Yes	0(0%)
***Greater than or equal to 25% drop in eGFR levels from baseline***	
No	66(100%)
Yes	0(0%)

**Table-II:** Pre- Post Ramadan Comparison.

Parameters	Pre-Ramadan	Post-Ramadan	P-value
Serum Creatinine (0.6-1.3mg/dl)	2.16±0.8	2.09±0.81	<0.0001
Serum Sodium (136-145 mEq/L)	139.42±2.33	139.76±1.82	0.247
Serum Potassium (3.5-5.1 mEq/L)	4.07±0.4	4.02±0.29	0.25
Serum Chloride (98-107 mEq/L)	105.95±3.46	107.11±2.89	0.039
Serum Bicarbonate (22-30 mEq/L)	21.88±2.56	21.95±2.16	0.783
eGFR	38.05±14.92	40.41±17.57	0.066
Systolic BP (mm Hg)	131.36±12.14	125.09±7.23	<0.0001
Diastolic BP (mm Hg)	76.21±6.51	72.42±4.58	<0.0001
Uric Acid (2.7-6.8 mg/dl in female) (3.5-7.2 mg/dl in male)	6.38±1.54	5.97±0.98	<0.0001

Data presented as mean±SD; P-value <0.05 considered to be statistically significant.

Out of 66 patients, 38 were diabetic and 28 were non diabetic. Baseline characteristics of both diabetic and non-diabetic subjects were almost similar except that only patients with CKD stage-3a, 3b and stage-4 were present in non-diabetic group as compare to diabetic group in which patients with stage-2 and stage-5 were also included (Table-III). Statistically significant improvement in pre and post Ramadan values was found in systolic and diastolic blood pressure, serum creatinine and uric acid levels in both diabetic and non-diabetic group (Table-IV).

**Table-III:** Characteristics of DM and Non-DM subjects.

Parameters	DM	Non-DM	P-value
n	38	28	
Age (years)	59.45±9.48	57.68±15.21	0.563
Male	20(52.6%)	14(50%)	0.833
Female	18(47.4%)	14(50%)
***CKD stage of baseline***			
2	3(7.9%)	0(0%)	0.255
3a	11(28.9%)	12(42.9%)	
3b	8(21.1%)	6(21.4%)	
4	13(34.2%)	10(35.7%)	
5	3(7.9%)	0(0%)	
Duration of CKD	4.86±2.26	4.74±2.86	0.853

Data presented as mean±SD or n(%); P-value<0.05 considered to be statistically significant.

**Table-IV:** Pre-Post Ramadan Comparison in DM /Non-DM subjects.

DM (n=38)	Pre-Ramadan	Post-Ramadan	P-value
Serum Creatinine	2.22±0.84	2.14±0.86	<0.0001
Serum Sodium	139.29±2.32	139.55±1.66	0.489
Serum Potassium	4.13±0.43	4.08±0.33	0.329
Serum Chloride	106.18±3.22	106.71±3.08	0.476
Serum Bicarbonate	21.16±2.4	21.53±2.02	0.138
eGFR	37.25±15.86	40.52±19.96	0.141
Systolic BP	129.47±11.38	125.82±6.97	0.028
Diastolic BP	75.79±6.42	73.03±5.14	0.035
Uric Acid	6.35±1.44	5.89±0.9	0.001

***Non-DM (n=28)***	***Pre-Ramadan***	***Post-Ramadan***	***P-value***

Serum Creatinine	2.07±0.76	2.01±0.75	0.005
Serum Sodium	139.61±2.36	140.04±2.01	0.343
Serum Potassium	3.98±0.35	3.94±0.21	0.527
Serum Chloride	105.64±3.79	107.64±2.56	0.019
Serum Bicarbonate	22.86±2.48	22.54±2.24	0.565
eGFR	39.14±13.76	40.26±14.06	0.004
Systolic BP	133.93±12.86	124.11±7.58	<0.0001
Diastolic BP	76.79±6.7	71.61±3.61	0.001
Uric Acid	6.41±1.68	6.07±1.09	0.03

Data presented as mean±SD; P-value<0.05 considered to be statistically significant.

## DISCUSSION

We found that Ramadan fasting inpatients with stable CKD both diabetic and non-diabetic did not result in any significant harmful effect on renal functions. Contrary to common belief, statistically significant improvement was observed in pre and post Ramadan values of systolic and diastolic blood pressure, serum creatinine, eGFR and uric acid eGFR is considered as an important factor by the nephrologists in assessing the impact of fasting on kidney functions and hence usually they do not advise fasting to patients who have CKD stage-4 and above.^8^

In this study mean pre-Ramadan eGFR was 38.05±14.92 while post Ramadan it was observed to be 40.41±17.57. Worsening in eGFR was not observed in any CKD stage in both diabetics and non-diabetic patients. Similar observation was seen in an another study done in local population which showed that there is no change in eGFR ,serum creatinine and proteinuria was documented in their study of 36 patients.^9^ Another study by Kara et al. concluded that fasting is not associated with deterioration in renal status of CKD patients.^10^ However, few studies have reported worsening of eGFR in patients with CKD stage-3 or above after fasting.^11^,^12^ Dehydration due to the inappropriate fluid intake during Ramadan fasting is a potential risk factor for rise in serum creatinine.

We observed that serum creatinine remained the same before and after Ramadan. In a study done by Dogan et al, increase in serum creatinine was observed in patients with CKD stage-3-5 at the end of the 1^st^ week of Ramadan. According to the authors use of diuretics and angiotensin-aldosterone blockers could be the reason of this deterioration in kidney functions.^13^ But in our study we haven’t observed any association of diuretics or Angiotensin aldosterone blockers with worsening of renal functions during Ramadan fasting. This contradictory finding may be due to the role of pre Ramadan assessment, Ramadan specific education as well as modifications of drug dosage and timings prior to Ramadan.

Previous studies have clearly demonstrated the role of pre-Ramadan assessment in safe fasting during Ramadan.^14^,^15^ Patients with both CKD and diabetes are considered at risk to develop potential complications from fasting specially those who live in hot climatic regions and with prolonged hours of fasting like in summer. Therefore, IDF DAR guidelines do not recommend fasting in diabetic patients with chronic kidney disease. However, these guidelines are mostly based on expert opinion as statistically powered long term studies are not available in the subject.

We did not observe worsening of kidney disease in both diabetic and non-diabetic group rather we have seen statistically significant improvement in post Ramadan serum creatinine of diabetic patients which may be due to strict dietary compliance. Similarly, no worsening in clinical, biochemical parameters and cardiovascular events was found in diabetic patients having moderate CKD in a survey done in east London.^16^

Hypertension was found to be a predictive factor for developing AKI in CKD patients who fasted in Ramadan which in turn put a bad impact on CKD progression.^17^ Our study showed that fasting in Ramadan is associated with reduction in systolic and diastolic BP in both diabetics and non-diabetic subjects. Similar observation was seen in meta-analysis done by Al-jafar et al.^18^ However, in their sub group analysis there was no significant difference in systolic and diastolic BP of CKD patients which is opposite from our study findings as we have found significant reduction in BP of CKD patients as well.

Due to the high content of potassium in dates, CKD patients are usually advised to avoid date consumption in Ramadan due to the risk of hyperkalemia. Breaking the fast with one date only may help in keeping the potassium levels in check. We did not observe any harmful impact of fasting on sodium, potassium, chloride and uric acid levels which is consistent with findings of another study which reported similar results.^19^ In our study we found statistically significant improvement in pre and post Ramadan readings of blood pressure, uric acid and serum creatinine in both diabetic and non-diabetic group.

It should be noted that most guidelines about fasting in patients with CKD stage-3 and above categorize them as high risk group for developing worsening of renal outcomes from fasting. Therefore, clinicians need to stratify patients with CKD on case to case (individual) basis so that potential risks about fasting can clearly be communicated to those who intend to fast despite medical advice. If patients still insist on fasting then proper counselling, providing dietary advice, modification in drug dosage and timings as well as educating about frequent self-monitoring of blood glucose and most importantly when to break the fast should be part of pre -Ramadan assessment.

### Limitations

Although the patients included in our study are from multiple centers and have multiple follow ups i.e. before, during and after Ramadan, however few limitations are there. The study has been conducted in a particular geographical area with specific climatic conditions and have small sample size. Results may vary if studies will be done on large population size and in different geographical areas having difference in climatic conditions with prolonged duration of fasting hours.

## CONCLUSION

Our study shows no worsening of renal functions in both diabetic and non-diabetic patients with stable CKD who intended to fast., However our sample size is small and further large scaled studies are needed to reassure safety of fasting in diabetic and non-diabetic patients with stable chronic kidney disease during Ramadan.

### Authors Contribution:

**AH:** Designed, did data collection, statistical analysis & manuscript writing, is responsible for integrity of research.

**MR:** Conception, design, manuscript writing and revised it critically and manuscript writing.

**BR** and **AS:** Did data collection, review and final approval of manuscript.
